# Meta-analysis and systematic review of factors biasing the observed prevalence of congenitally missing teeth in permanent dentition excluding third molars

**DOI:** 10.1186/2196-1042-14-33

**Published:** 2013-10-01

**Authors:** Vahid Rakhshan

**Affiliations:** Department of Dental Anatomy and Morphology, Dental Branch, Islamic Azad University, PO Box 19585-175, Tehran, Iran

**Keywords:** Congenitally missing teeth (hypodontia), Permanent dentition, Prevalence, Sources of bias in the literature

## Abstract

No meta-analyses or systematic reviews have been conducted to evaluate numerous potential biasing factors contributing to the controversial results on congenitally missing teeth (CMT). We aimed to perform a rather comprehensive meta-analysis and systematic review on this subject. A thorough search was performed during September 2012 until April 2013 to find the available literature regarding CMT prevalence. Besides qualitatively discussing the literature, the meta-sample homogeneity, publication bias, and the effects of sample type, sample size, minimum and maximum ages of included subjects, gender imbalances, and scientific credit of the publishing journals on the reported CMT prevalence were statistically analyzed using *Q*-test, Egger regression, Spearman coefficient, Kruskal-Wallis, Welch *t* test (alpha = 0.05), and Mann-Whitney *U* test (α = 0.016, α = 0.007). A total of 111 reports were collected. Metadata were heterogeneous (*P* = 0.000). There was not a significant publication bias (Egger Regression *P* = 0.073). Prevalence rates differed in different types of populations (Kruskal-Wallis *P* = 0.001). Studies on orthodontic patients might report slightly (about 1%) higher prevalence (*P* = 0.009, corrected α = 0.016). Non-orthodontic dental patients showed a significant 2% *decline* [*P* = 0.007 (Mann-Whitney *U*)]. Enrolling more males in researches might significantly reduce the observed prevalence (Spearman ρ = -0.407, *P* = 0.001). Studies with higher minimums of subjects' age showed always slightly less CMT prevalence. This reached about -1.6% around the ages 10 to 13 and was significant for ages 10 to 12 (Welch *t* test *P* < 0.05). There seems to be no limit over the maximum age (Welch *t* test *P* > 0.2). Studies' sample sizes were correlated negatively with CMT prevalence (ρ = -0.250, *P* = 0.009). It was not verified whether higher CMT rates have better chances of being published (ρ = 0.132, *P* = 0.177). CMT definition should be unified. Samples should be sex-balanced. Enrolling both orthodontic and dental patients in similar proportions might be preferable over sampling from each of those groups. Sampling from children over 12 years seems advantageous. Two or more observers should examine larger samples to reduce the false negative error tied with such samples.

## Review

### Introduction

Congenital missing of teeth (CMT) or dental agenesis is a common dental abnormality, in which some dental buds fail to develop, leaving an empty space in the arch which causes numerous complications [1–19]. In most countries, out of every 10 to 20 individuals, at least one suffers from agenesis of at least one or two permanent teeth [1, 3–13, 16–109]. It is of importance since not only it is very frequent (as the most common dental anomaly) [1–14], but also it needs difficult and expensive treatments [19, 105, 110, 111].

Considering the very high prevalence of CMT [1–14], its serious complications on esthetic and function [2–4, 15–19, 56, 75, 77, 83, 86, 89],[97, 100, 105, 112–117], and its challenging and costly multidisciplinary treatments [19, 105, 110, 111], studying it seems necessary for many fields. These involve public health, health insurance companies, anthropology, and of course multidisciplinary clinical practice (orthodontics, prosthodontics, pediatric dentistry, surgery, and general dentistry) [40, 105, 111, 117, 118].

The results pertaining to CMT are quite controversial. Although ethnicity accounts for a part of the debate [117] (V Rakhshan, unpublished work), a major source of dispute is the existence of different biasing factors in different reports. For example, enrolling younger subjects might increase the chance of encountering 'delayed’ tooth eruptions and mistakenly counting the empty spaces as CMT [18, 117]. Additionally, it is possible that researchers might tend to report the lower CMT prevalence in larger samples and vice versa [18, 117]. These and other biasing factors should be determined and avoided. Nevertheless, only the aforementioned two examples are meta-analyzed before, and in a small pool of 33 studies [117]. That meta-analysis confirmed the role of sample size but did not find any differences between studies with minimum ages of subjects older or younger than 7 years. They did not evaluate any other minimum ages or address the potential lack of test power due to their small sample. No other biasing factors have been analyzed thus far.

Moreover, recent studies are limited to carry out the sampling almost only from orthodontic and dental patients. This is possibly due to ethical concerns tied to X-ray exposure without any treatment need [18, 116]. Such samples are assumed to result in overestimation of CMT. However, it is not known whether this assumption is correct [86]. Besides, many studies do not sample an equal number of males and females. Females might somehow show higher CMT prevalence [10, 11, 17, 35, 36, 50, 82, 83],[91, 100, 117, 119] (V Rakhshan, unpublished work). Therefore, a question is whether enrolling more females can noticeably bias the CMT result (and if so, to which extent). Another possibility is that older patients might not remember their history of extraction, and therefore some extracted teeth might be considered as missing. Knowledge of the effects of these factors is of importance. However, no meta-analyses, systematic reviews, or even narrative reviews have explored these. The literature consists only of two rather narrow-scoped, small-sampled meta-analyses and two partially narrative literature reviews on CMT [115, 117, 120]. Hence, the aim of this study was to address the potential sources of bias of CMT prevalence, using more comprehensive search strategies and a larger sample.

### Materials and methods

During September 2012 until April 2013, the author extensively searched for the keywords 'congenital missing of teeth’, 'CMT’, 'hypodontia’, 'oligodontia’, 'anodontia’, 'agenesis’, and 'prevalence’ [117], and combination of these words as well as their synonyms (for example, replacing 'missing’ with 'absence’, 'absent’, etc.; Table 1). Three Internet search engines [Pubmed, Thomson Reuters ISI Web of Science (WoS), and Google Scholar] were used by the author to search for relevant scientific articles (V Rakhshan, unpublished work).

**Table 1:** **Number of search results**

Keyword	Pubmed	Web of Science	Google Scholar	Total
Variations of 'congenitally missing teeth^a^’	399	95	3,133	3,627
Hypodontia or anodontia or oligodontia	3,345	940	12,300	16,585
'Dental aplasia’ or 'dental agenesis’ or ’tooth aplasia’ or 'tooth agenesis’	353	398	3,300	4,051
Congenitally missing teeth^a^ and prevalence	102	27	1,630	1,759
(Hypodontia or anodontia or oligodontia) and prevalence	490	170	3,560	4,220
(Dental aplasia or dental agenesis or tooth aplasia or tooth agenesis) and prevalence	100	106	1,560	1,766
Total	4,789	1,736	25,483	32,008

^a^Including the search results for congenital missing of teeth, congenital absence of teeth, and congenitally absent teeth.

Reports dealing with patients suffering from craniofacial syndromes or developmental disorders were excluded. Those including only primary dentition were as well excluded. The reports taking the rates of third molar missing cases (without presenting any information to filter third molars out) were excluded (V Rakhshan, unpublished work).

The inclusion criteria were as follows:

- The presence of an English abstract or an abstract readable by the author; or when other studies cited local papers and provided useful information from earlier non-English papers on CMT in permanent dentition excluding third molars. The articles cited within the full texts were used after making sure that they meet the inclusion criteria.
- The sample was representative of the underlying general population.
- Diagnosis of dental agenesis was based on a radiographic examination (if not stated otherwise in them or in the citing articles).
- Agenesis of third molars was excluded [117].
- Data pertaining to at least one of the biasing factors of interest alongside CMT prevalence were present (V Rakhshan, unpublished work).

Not all the studies contained all the information simultaneously. As far as at least the prevalence and one biasing factor were reported by a study, it was used in this summary.

The texts of all the available full articles and the abstracts were read carefully by the author. Whenever the necessary data were available (regardless of analyses performed by articles' authors), all the percentages were recalculated. Raw data of one of them were inconsistent, as the two tables presenting the raw data did not match completely [88]. Every available English full text on CMT excluding third molars was read for at least twice. If there was a full text in another language, collecting any useful data piece was tried by means of translators or by digging for the presented numbers or English figure legends, etc. within the article. Some of those articles referred to other studies or reported briefly the previous studies. After making sure that those previous studies do not include third molars (or if they do, it is possible for us to filter or recalculate only information regarding hypodontia excluding third molars), they were included in this summary. Whenever it is possible to recover the raw data from the information, the percentages presented were recalculated and at many points fixed before reporting. Also, many studies had not reported some of the key elements. If possible, their raw data were carefully recovered from the combination of their text, graphs, and tables (V Rakhshan, unpublished work).

#### Statistical analysis

The reported prevalence rates in epidemiological studies were compared with those of orthodontic or dental patients, using a Kruskal-Wallis test and a Mann-Whitney *U* test. The level of significance was adjusted for the Mann-Whitney *U* test to 0.016, using the Bonferroni correction method for multiple comparisons. The association of publication year with reported CMT percentages was assessed in the literature and in the literature on Caucasians only, using a Spearman correlation coefficient. Also, the correlation between CMT prevalence and the ratio of enrolled male and female subjects in different studies was assessed using a Spearman coefficient. The Spearman coefficient was also used to analyze the potential association of sample size and CMT prevalence. Additionally, the scientific credit of each article was determined by checking its abstraction in Pubmed and Web of Science databases or both (the score 0 for none, 1 for each, and 2 for both); the correlation between the CMT and scores of the articles was evaluated using the Spearman coefficient. The potentially biasing role of minimum and maximum ages of enrolled subjects in different studies was assessed using the Spearman correlation coefficient. We tried to find also any cutoff age after which a significant decrease in CMT could be possibly observed. This was performed by comparing studies with minimum ages less or greater than each cutoff using a Welch *t* test. The same test was used in the same fashion to possibly find a cutoff point for the maximum age.

## Results

About 24,000 studies were initially found via various search engines which this reduced to about 8,000 more relevant results (Table 1) and then to about 2,500 studies without counting the repetitions in different search engines. Searching was updated during the study period to find newly emerged articles, and one new relevant study was published in March 2013. The excluded studies were the duplicated ones (in different search websites), those with no reference to the exclusion of third molars or syndromes, or those pertaining to other aspects of CMT without presenting at least CMT prevalence in permanent dentition and at least one biasing factor. If at least they had graphs or tables from which the CMT prevalence could be recovered and/or the data allowed us to exclude third molars and syndromes, the prevalence would be calculated manually. All the procedures were done for at least twice by one examiner (and for some articles, more than two times). Finally, 111 reports were included in this review (Table 2) [1, 3–13, 16–109, 121] (V Rakhshan, unpublished work).

**Table 2:** **The reported frequencies on missing of all permanent teeth except the third molars**

Year	Country	Type	Prevalence	Year	Country	Type	Prevalence	Year	Country	Type	Prevalence
1936	Switzerland	SC	3.4	1974	Canada	SC	7.4	2001	Kenya	OP	6.3
1939	-	-	2.3	1976	Sweden	SC	7.4	2001	Hungary	OP	15.7
1943	USA		2.8	1977	Iceland	SC	7.9	2001	Korea	SC	8.0
1949	Japan	St	15.9	1977	Sweden	SC	7.4	2002	Norway	PuDP	4.5
1951	Japan	SC	5.6	1977	Japan	-	8.6	2002	Iraq	OP	8.9
1954	Japan	SC	8.7	1979	USA	SC	7.4	2002	S Arabia	PeDP	3.6
1955	Japan	DS	1.4	1980	Denmark	SC	7.8	2003	Mexico	OP	2.7
1956	Sweden	SC	6.1	1980	Denmark	SC	7.7	2005	Slovenia	OP	11.3
1956	USA	DP	3.7	1987	Hong-Kong	SC	6.9	2006	Japan	OP	10.1
1959	Sweden	-	7.4	1988	Japan	OP	9.9	2006	Jordan	DP	5.5
1961	USA	DP	5.2	1989	Malaysia	SC	2.8	2006	Hungary	OP/PeDP	14.7
1963	Norway	SC	4.5	1989	Australia	DP	6.4	2007	Turkey	OP	7.5
1963	Austria	SC	9.6	1989	Italy	DP	5.2	2007	Turkey	OP	2.8
1963	Japan	SC	6.6	1989	USA	PeDP	7.8	2008	Italy	OP	9.5
1964	USA	DP	5.1	1989	S Arabia	DP	2.2	2008	Japan	PeDP	9.8
1965	Israel	SC	0.3	1989	Ireland	-	11.7	2008	Brazil	PeDP	4.8
1966	UK	OP	4.3	1989	Czechoslovakia	-	4.1	2008	Korea	OP	11.2
1963	Canada	DP	4.2	1990	S Arabia	SC	4.0	2009	Spain	OP	6.5
1966	USA	–	6.5	1990	Japan	PeDP	16.2	2009	Turkey	DP	73.4
1967	USA	SC	3.8	1990	Thailand	OP	8.6	2010	Pakistan	OP	9.0
1967	USA	SC	4.1	1990	Australia	DF	6.3	2010	India	DP	0.1
1968	Australia	SC	5.9	1990	Ireland	OP	11.3	2010	Iran	DP	9.0
1968	Denmark	SC	6.1	1990	Yugoslavia-Istria	OP	6.3	2010	Turkey	OP	4.6
1970	USA	St	3.5	1990	Yugoslavia-Slavonia	OP	2.3	2010	Spain	PHS	7.3
1970	USA	St	3.6	1991	Italy	DF	3.6	2010	Iran	OP	9.1
1971	Finland	SC	8.0	1992	Japan	OP	10.9	2010	S Arabia	OP	7.0
1971	-	-	5.5	1992	-	OP	5.3	2010	Turkey	DP	1.5
1971	Sweden	SC	6.3	1993	Norway	SC	6.5	2011	Korea	OP	11.3
1972	Japan	SC	9.2	1994	Germany	OP	8.1	2011	Korea	DP	5.7
1972	Japan	OP	11.0	1995	Japan	PeDP	2.8	2011	India	DP	4.5
1973	Israel	-	4.6	1996	Mexico	OP	6.3	2011	Germany	OP	13.1
1973	Denmark	–	8.2	1997	Iceland	SC	4.3	2012	Iran	OP	9.0
1973	Norway	SC	10.1	1997	Estonia	SC	14.0	2012	India	SC	0.3
1973	Sweden	SC	6.1	1998	China	-	7.3	2012	Turkey	PeDP	6.2
1974	UK	SC	4.4	1999	S Arabia	DP	4.2	2012	Portugal	DP	6.1
1974	Switzerland	SC	7.7	1999	Brazil	OP	6.3	2012	S Arabia	DP	4.7
1974	Norway	SC	6.8	1999	Japan	OP	9.4	2012	Iran	OP/DP	10.9

DS, dental student; OP, orthodontic patients; SC, schoolchildren; PuDP, public dental patients; PeDP, pediatric dental patients; DF, defense force recruits; PHS, attendees to the primary health services; DP, dental patients other than orthodontic patients.

A single study was published in two different journals (one indexed in Pubmed and the other not indexed in any accredited databases), in the same year, without showing any noticeable difference in the content (thus both were considered as a single report) [95, 122]. The sample of Gabris et al. [78] seemed to be used as a subsample in their other study [85], although it is not known for sure as they did not suggest it. Syndrome cases were removed by the author from the study of Galluccio and Pilotto [87]. Cleft palate cases were excluded by the author, and the prevalence was recalculated for the study of Behr et al. [105]; however, four patients among their remaining cases were syndromic. From the study of Ghaznawi et al. [74], third molars were excluded by the author. Osuji et al. [81] reported only the missing of teeth anterior to molars. The sample of Sheikhi et al. [121] and Gabris et al. [85] were composed of both dental and orthodontic patients but the ratio of each was not known. A research had reported the maximum age as both 25 and 53 in different parts of the article [121]. Their maximum age was not used in analyses.

### Homogeneity of metadata

Data were heterogeneous (*Q* = 7287.8, *I*^2^ = 98.5, *P* = 0.000) meaning that besides the sampling error due to the differences in the samples, other factors accounted for the different results as well.

### The biasing role of sample types

**Figure 1:**
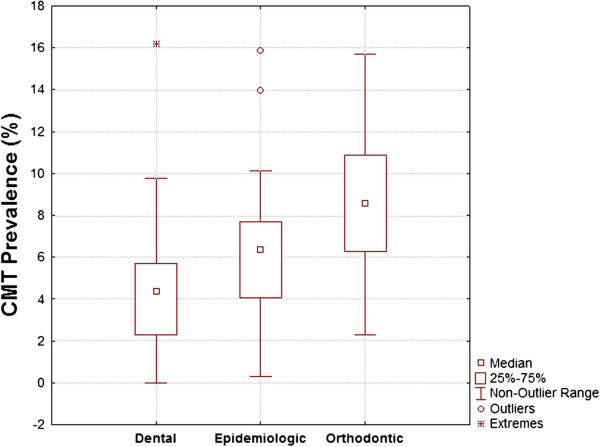
**Box plots for different CMT prevalence rates in three population types.** Taking into analysis 43 epidemiological studies, 32 studies of orthodontic patients, and 29 studies on dental patients. Pediatric dental patients are categorized as dental patients.

Studies on orthodontic patients might report prevalence rates about 1% greater, while those of dental patients might report prevalence rates about 2% smaller than epidemiological studies (Figure 1). The Kruskal-Wallis test showed that the difference between reports on epidemiological samples versus orthodontic and dental patients is significant (*P* = 0.000). The Mann-Whitney *U* test showed that the pairwise comparisons were as well significant between epidemiological samples with dental patients (*P* = 0.007) and orthodontic patients (*P* = 0.009), and also between orthodontic and non-orthodontic dental patients (*P* = 0.000, corrected α = 0.016).

### Compositions of samples in terms of sex balance

The Spearman coefficient showed a significant correlation between CMT prevalence and male/female ratios (calculated by dividing the number of male subjects by females subjects) (*n* = 62, *ρ* = -0.407, *P* = 0.001).

### Minimum age of included subjects

**Figure 2:**
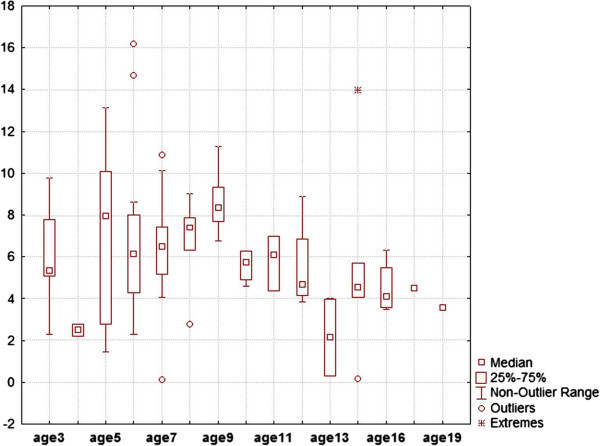
**Box plots illustrating CMT of studies with different minimum ages of subjects.**

The minimum age was not significantly correlated with CMT (*n* = 79, *ρ* = -0.131, *P* = 0.255, Figure 2). Each minimum age was used as a cutoff to examine whether at any specific minimum age, there is a possibility to see significant differences between the CMT reported by studies adopting subjects younger and older than that age (Table 3). The comparisons were done using an unpaired *t* test with Welch correction. An almost steady reduction was seen for most of the cutoffs, between studies adopting subjects with minimum ages less than each cutoff and those enrolling minimum ages over that cutoff. At the age of 10 (comparing <10 with ≥10), the CMT prevalence was significantly different between studies with minimum ages set at less than 10 and those at 10 or older (*P* = 0.018). This also happened at the age 11 (*P* = 0.028) and 12 (*P* = 0.033). The highest difference was observed at the age of 13, but it was only marginally significant (Table 3).

**Table 3:** **Results of Welch** ***t*** **test comparing studies' CMT results (%) according to various minimum ages**

Cutoff age (year)	<Cutoff		≥Cutoff			
	***N***	Mean (%)	***N***	Mean (%)	Difference	***P*** value
6	13	5.85	64	6.27	0.42	0.704
7	28	6.41	49	6.08	-0.33	0.687
8	37	6.39	40	6.02	-0.36	0.62
9	42	6.42	35	5.93	-0.49	0.49
10	50	6.77	27	5.14	-1.64	*0.018*
11	54	6.68	23	5.06	-1.63	*0.028*
12	57	6.64	20	4.94	-1.70	*0.033*
13	64	6.52	13	4.60	-1.92	0.074
14	66	6.39	11	5.05	-1.34	0.239
16	71	6.34	6	4.50	-1.84	*0.011*

***N*** number of studies.

### Maximum age of included subjects

The Spearman coefficient showed no significant correlation between the maximum ages and CMT prevalence (*n* = 73, *ρ* = -0.003, *P* = 0.978). Ages 15, 20, 25, 30, 35, 40, 45, and 50 were considered potential cutoffs. The comparison of the results of studies with maximum ages of patients less than or above these cutoff ages showed no significant differences. An outlier study with CMT = 71.38% was in the analysis. After removing the outlier, the rest were re-analyzed. Although the results were nonsignificant, at the cutoff maximum age of 45 years, the *P* value showed a peak from values >0.8 to 0.242, and the difference changed from about 0.5% to 2% but still nonsignificant (i.e., the studies enrolling older ages showed about 2% greater CMT rates).

### Sample size as a biasing factor

The sample size was significantly and negatively correlated with CMT (*n* = 109, *ρ* = -0.250, *P* = 0.009).

### Publication bias

**Figure 3:**
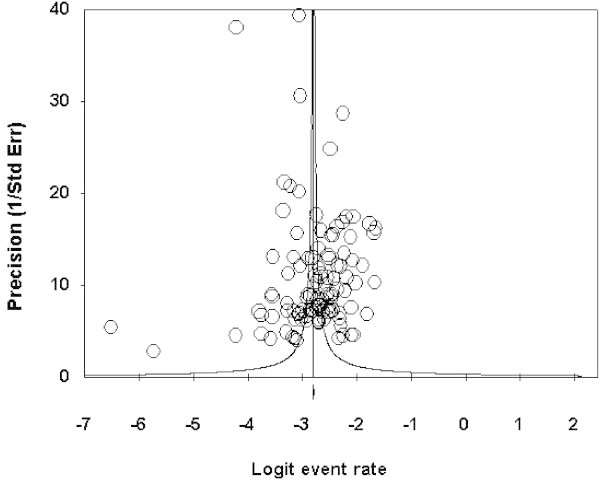
**A rather symmetrical funnel plot (with some outliers) of study precisions against effect sizes.**

By analyzing the potential asymmetry in the funnel plot (Figure 3), the Egger regression test pointed to a marginally significant publication bias (intercept = 2.81, SE = 1.55, 95% CI = -0.26 to 5.90, *P* = 0.073).

### Correlation between scientific level of journals and the reported CMT prevalence

The one study published in two different journals was considered as Pubmed-indexed. The outlier study of Peker et al. [91] was excluded from the analysis. There was no significant link between the credit of the article and the CMT prevalence reported (*n* = 107, *ρ* = 0.132, *P* = 0.177). The same analysis was done for studies published after 1990, and the association was still nonsignificant (*n* = 54, *ρ* = 0.182, *P* = 0.188).

## Discussion

The literature consists of so many studies on the prevalence of CMT in permanent dentition (excluding third molars) among different populations [1, 3–13, 16–109, 114, 123–125]. The results are extremely controversial, ranging from 0.1% to 16.2% and varying considerably in many countries. However, they mostly revolved around 7% [mean = 6.72 ± 3.28, *n* = 110 (excluding one unreliable outlier showing 74%)] [1, 3–13, 16–109]. The different rates reported could be explained by the ethnic backgrounds [4, 18, 54, 100, 112, 117, 118] and variations in the samples with respect to sample sizes, types, and other biasing factors [2, 3, 18, 86, 117]. Another (unpublished) meta-analysis of this author shows that CMT may happen more in Yellow race and perhaps in Europeans, while it is less common in the west of Asia and America. Also this anomaly is not being increased in epidemiological samples through time, although inclusion of dental patients in recent studies is biasing this results (V Rakhshan, unpublished work). The variations are not only a reflection of ethnicity, but can occur due to biasing factors as well. Discerning the responsible sources of bias can benefit future studies.

### Should studies be sex-balanced?

**Figure 4:**
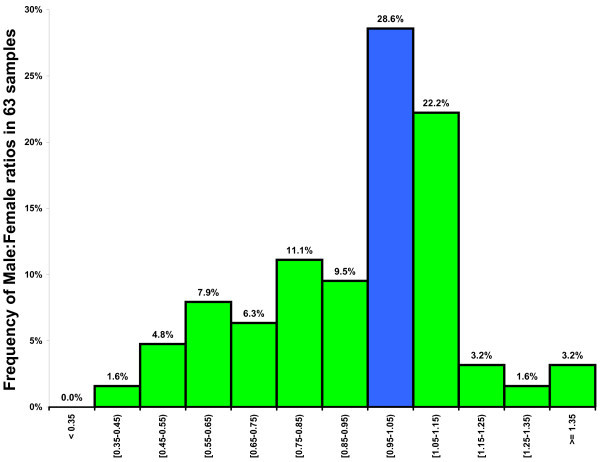
**Frequency distribution of 63 ratios of enrolled male and female subjects.** Frequency distribution (%) of 63 ratios of enrolled male and female subjects, excluding one study with a male/female ratio of 4.21 [16] and two studies on only males [61, 66]. The blue bar is indicative of gender-balanced samples. Ratios greater than 1 show a gender imbalance in favor of males, and those smaller than 1 denote more females.

Samples not balanced in terms of gender can bias the result, as CMT is more likely to occur in females [10, 11, 17, 35, 36, 50, 82, 83],[91, 100, 117, 119] (V Rakhshan, unpublished work). Our statistical analysis confirmed the role of the male/female ratio in affecting the prevalence. This ratio was considerably different from 1.0 in many studies (Figure 4). Such designs should be avoided in the future.

### Do minimum and maximum ages of included subjects matter?

Calcification of teeth usually completes until the age of 9 years. Sometimes calcification of premolars is delayed [18, 69, 74, 86]. The absence of a premolar in a radiograph cannot be certainly regarded as missing until this age, or the age of 10 years [117], particularly in boys [18, 77, 86, 98, 102]. Therefore, patients should be assessed in the third and fourth dental stages (DS), during which canines/premolars erupt and occlude with their antagonist teeth, respectively [102]. Inclusion of DS 1 and DS 2 (eruption and occlusion of incisors) and earlier stages should be avoided [102]. Some evidences clearly confirm the decrease of the observed CMT prevalence in a single population after 2 years (7-year old children compared to themselves at the age of 9) [18, 48, 86]. This can compromise studies which have enrolled subjects as young as 5 [100, 126] or 7 years old [4, 121]. It probably causes high prevalence of second mandibular premolar missing (and also general CMT) [3, 10, 17, 48, 117]. Second premolars can develop long after what it would be ordinarily expected, reducing the degree of certainty [3]. Therefore, some authors have recommended exclusion of children younger than 7 [3], 9 or 10 years old [18, 77, 86, 98, 117], or even under 16 [18]. Nevertheless, some authors refuted this by following a population of 7-year-old children evaluated first in 1976 until 1990 [4]. They found that only one out of 739 subjects showed late mineralization of teeth. They concluded that samples with minimum ages lower than 9 might as well lead to reliable results [4]. Also Varela et al. [75] assessed children under 10 years old with dental agenesis some years later and found no tooth germs in the place of previously found missing teeth [75].

Polder et al. [117] evaluated the prevalence reported in 33 studies stratified into those with older and younger than a minimum inclusion age of 7 years old. They did not find a significant difference (*P* = 0.42). The same happened in our study, when ages 6, 7, 8, and 9 were the cutoff ages. Nevertheless, we investigated more ages as cutoffs. The post-cutoff decrease reached the level of significance when it came to the ages 10, 11, and 12. Therefore, it seems that by enrolling people older than these ages, overestimation of CMT (due to false positive diagnosis of missing) is considerably reduced and these can be possibly considered of the best minimum ages. However, at older ages such as 16 as well, significant differences emerged, which imply that false positive diagnosis might occur even until 16. Since the number of subjects at a minimum age set by each study is not known, the mentioned minimum age does not necessarily reflect that a considerable part of a sample are at that minimum age or around it. This can blur the link between the stated minimum age and CMT. However, there was still a reduction (even if nonsignificant) for almost all the cutoff ages. It validates the claim that there might be a share of false positive in younger ages (which can be generalized at ages 10 to 12). It might be recommended to avoid the inclusion of subjects younger than 12 or 13, in order to make sure an empty space seen in the radiograph at that age is almost only a case of missing, not a delayed tooth bud development. Of course sampling from older ages (such as older than 16) is better [18], but this has a trade off with the ease of finding proper test subjects.

It was hypothesized that perhaps enrolling older subjects might increase the false positive error due to the addition of the extracted teeth into the sample. Two reasons are imaginable for the lack of significance. First, many studies enrolled only full sets of dentition (regardless of patients' age). Second, a few studies had high maximum ages.

### Do orthodontic or other dental patients necessarily show 'greater’ missing rates?

It is suggested that children with CMT may be more prone to visit orthodontists compared to individuals without missing, which this can affect the findings [5, 17, 18, 37, 86, 125, 127]. However, Sisman et al. [86] showed that CMT prevalence is similar for orthodontic patients and epidemiological populations. On the other hand, our analyses showed that studies of orthodontic and other dental patients might have a slight but still statistically significant error (Figure 1). It was interesting that the prevalence rates reported by studies on dental patients (except orthodontic patients) were unexpectedly smaller than epidemiological samples. It seems that enrolling orthodontic patients should be accompanied with enrolling dental patients in similar ratios, in order to possibly offset or reduce their biasing roles at opposite directions. However, this needs further studies.

### Do smaller studies report higher prevalence?

It might be plausible that when fewer research subjects are available, there might be a bias to catch more cases of interest. Also in larger studies, it is possible that exhausted researchers overlook some of existing cases. As well, small studies with low prevalence of dental agenesis might be less likely to be submitted or accepted for publication [117]. Therefore, smaller studies might show larger prevalence rates. Our findings confirmed that the sample size correlates negatively with the CMT. It is not easily known, however, whether the smaller prevalence rates are closer to reality or the greater ones, because there is no gold standard to estimate the sensitivity and specificity of these two and conduct receiver-operator curves. However, it can be recommended that sufficiently large samples (based on *a priori* power calculation) should be assessed by two or more observers. This way, the advantages of a larger sample remain while the odds of false negative errors reduce.

### Do greater CMT prevalence rates have better chances of being published?

It was suggested that smaller samples with smaller CMT rates might have less chances to be published [117]. Our analysis could not verify this, although a marginally significant asymmetry was detected in the funnel plot. This lack of significant publication bias could be due to the rather more extensive literature search done in this study, which allowed many non-English and average articles to be pooled. Therefore, the author tried to assess whether such a trend exists. A higher chance of publication can increase the publication likelihood in acclaimed journals. So if better journals published higher CMT prevalence rates, the above assumption might be confirmed. Also since many studies published in the previous century were reported in accredited but not indexed journals, we repeated this analysis for studies published after 1990 (when the Pubmed and WoS databases were more popular in dental literature). Nevertheless, although the correlation increased slightly, it was still nonsignificant (in part due to the reduction in the new sample size). Therefore, we could not verify that if higher CMT prevalence rates have a greater chance of publication. It should be noted that although we tried to include local journals as well (if found by searching), there might be still many studies ruled out in this meta-sample due to the language bias.

### Other potential limitations and sources of bias

A limiting obstacle is the difficulty to accurately distinguish the absent tooth from adjacent similar teeth [102]. This is noticeable especially in the case of mandibular incisors when there are three incisors [3, 18, 102], for which meticulous examination of dental casts can be helpful [89, 102]. It can be more difficult when the other teeth have moved, and also when the image of the vertebrae is superimposed on the anterior mandible [3].

Clinical examinations may cover merely 70% of actual cases of CMT, and radiographic examination is always necessary [3, 18, 93]. However, X-ray exposure for whatever purposes except treatment is unethical (even screening, since it is not effective [57]) [18, 116]. Besides, orthodontic materials are appropriate for diagnosing CMT [89]. Therefore, recent papers are enrolling only orthodontic or dental patients. Our analyses showed that orthodontic patients or dental patients might show about 1% to 2% error compared to epidemiological samples, but there seems to be no other choice. Some studies enrolled epidemiological populations but reduced the number of radiographs taken, by clinically examining the area at first and ordering X-ray exploration if in doubt. Nevertheless, this method might introduce sampling bias [117].

### Why should definitions be unified?

Another issue can be the variation in definitions. Specific terminologies are used to describe the nature of tooth agenesis. In general, the term hypodontia is most commonly used to describe the phenomenon of CMT [83]. Many other terms to describe a reduction in the number of teeth appear in the literature: oligodontia, anodontia, aplasia of teeth, congenitally missing teeth, absence of teeth, lack of teeth, and agenesis of teeth [83]. Hypodontia, oligodontia, and anodontia differ in terms of the number of missing teeth. Nevertheless, there is no clear consensus over the threshold differentiating hypodontia from oligodontia [3, 100, 114]. The categories used for defining oligodontia is the absence of more than three [16, 113], more than four [5, 89], more than five [3, 15, 48, 79, 91, 96, 100, 102],[105, 114, 117, 118, 128], more than six teeth [82, 83, 98, 104, 114], and even more than ten teeth [69, 129], always excluding the third molars [3]. Some authors did not define their threshold [1].

In some studies, the problem was beyond using different definitions. They basically confused the terms. For example, some investigators reported CMT under the name hypodontia [4, 19, 75, 77, 89, 90, 94, 98],[102, 106]. A study reported hypodontia as CMT [109]. Some even reported oligodontia cases as a part of hypodontia (again implying that hypodontia was considered CMT) [98, 102]. A study considered hypodontia as people with missing cases less than six teeth but more than two [105]. Some studies did not give any clear clue as if they were referring to CMT by hypodontia or not [4, 75, 106, 130]. In some of them, there were certain vague (but not official) remarks implying that perhaps the CMT was the case [4, 75, 130]. All of the above-mentioned variations can account for the high heterogeneity observed in this meta-sample.

It might seem that the percentages reported by the erroneous definitions should be similar usually, as oligodontia has a low frequency. For example, two studies which mistook CMT with hypodontia, showed only one subject with more than six absent teeth [4] or even not more than six missing teeth [77]. Therefore, their CMT results [77] might be pointing to hypodontia as well. Besides, since the prevalence of missing of four or more teeth is scarce, changing the threshold of hypodontia definition from >3 teeth to >6 teeth might lead to slight changes only.

Nevertheless, in some populations, it can still account for some more vivid changes. About 10% to 25% of hypodontia cases are individuals with more than two teeth missing [4–6, 8, 10, 13, 17, 18, 27, 36],[40, 41, 46, 48–50, 53, 54, 62, 76],[77, 79, 81, 86, 88, 89, 102, 106],[117, 125, 127]. About 5% of the population might have more than three missing teeth [89] or about 5.5% might have four and about 1.5% might have five absent teeth [86]. There can be about 6% and 5.4% prevalence of patients with four and five teeth missing, respectively in Japanese [3], about 7% prevalence for four missing teeth in Brazil [88], 7% prevalence of more than five missing teeth in Turkey [91], or 16% of more than five absent teeth in Germany [105]. Therefore, the unification of these terminologies as CMT instead of hypodontia etc., and using standardized templates to clearly define CMT and mild, moderate, and severe cases of CMT or hypodontia are necessary.

## Conclusions

Limitations of previous reports make comparisons difficult and at some points impossible. A need for a standardized template is perceived to unify methodologies and also enable researchers to assess this issue with the most possible details.

Future studies should use standardized cutoff values for defining CMT/hypodontia and also standard methods for sampling and diagnosis. Future meta-analyses should include studies on orthodontic/dental patients as well, since due to the ethical concerns regarding X-ray exposure, epidemiological studies are unlikely affordable. Sampling from orthodontic patients might be preferable over dental patients or pediatric dentistry patients. This is because CMT results of orthodontic samples were closer to the epidemiological samples. However, it might be recommended to sample from both simultaneously, as their roles in biasing the prevalence are opposite of each other. Studies should not enroll children younger than 12 years. There seems to be no limit over the maximum age at least as long as positive extraction history is an exclusion criterion. It is recommended to adopt sufficiently large samples (based on power calculations) because of its obvious advantages. But since observers might miss some cases in such samples, two or more examiners should judge large samples to reduce or eliminate the corresponding false negative error. There seems to be no strong publication bias for accepting higher CMT prevalence rates.

## Abbreviations

CMT: Congenital missing of teeth.
